# Urodynamic findings in children with primary refractory nocturnal enuresis: 10 years' experience of a tertiary center

**DOI:** 10.1002/hsr2.1626

**Published:** 2023-10-10

**Authors:** Farzaneh Sharifiaghdas, Behzad Narouie, Mohadese Ahmadzade, Hamidreza Rouientan, Darya Najafi, Mehdi Dadpour, Nariman Latifi, Hamideh Hanafi Bojd, Sobhan Sabzi

**Affiliations:** ^1^ Department of Urology, Shahid Labbafinejad Medical Center, Urology and Nephrology Research Center Shahid Beheshti University of Medical Sciences Tehran Iran; ^2^ Department of Urology Zahedan University of Medical Sciences Zahedan Iran

**Keywords:** enuresis, MNE, NMNE, urodynamic study, UDS

## Abstract

**Background/Aim:**

To identify correlations between urodynamic study (UDS) findings and urinary symptoms in children with refractory monosymptomatic and nonmonosymptomatic primary nocturnal enuresis.

**Materials and Methods:**

A total of 96 neurologically normal children were enrolled, 44 consecutive boys and 51 consecutive girls, aged 5–18 years, of whom 41 (38.8%) had refractory monosymptomatic nocturnal enuresis (MNE) and 55 (61.2%) had refractory non‐MNE (NMNE). We assessed the urodynamics of all children to detect any underlying bladder overactivity. A comparative analysis was carried out between the two groups of patients.

**Results:**

Detrusor overactivity (DO), low bladder capacity, low compliance, and increased postvoid residual (PVR) were identified in 70 (72.9%), 35 (36.5%), 43, and 76 (79.2%) patients, respectively. The mean bladder compliance was 21.66 ± 14.52 mL/cmH_2_O (2–75 cmH_2_O). Of the NMNE patients, 50 (90.9%) had abnormal urodynamic findings, while 40 (97.5%) had abnormal urodynamic findings in the MNE group. There was a statistically significant relationship between NMNE and both increased PVR and abnormal voiding patterns. Both high PVR and DO were significantly associated with obstructive urinary symptoms. Constipation and history of urinary tract infection (UTI) did not significantly correlate with UDS abnormality (*p* = 1.0 and *p* = 0.49, respectively).

**Conclusion:**

There was a high prevalence of bladder function disorders in both refractory MNE and NMNE patients in our study. This included small functional capacity, low bladder compliance, and marked DO. A nocturnal enuresis may be the only presenting symptom, however, it may be associated with bladder overactivity, UTI, and constipation; the UDS findings may aid in guiding the assessment and treatment of children suffering from primary refractory nocturnal enuresis and its association with bladder and bowel symptoms.

## 1 INTRODUCTION

The International Children's Continence Society (ICCS) defined nocturnal enuresis (NE) as an intermittent incontinence symptom that occurs during periods of sleep. Refractory NE is defined as less than 50% improvement in symptoms with treatment for at least 3 months.^1^ Approximately 3.9−18.7% of children and almost 2% of adults are affected by primary NE on average three times per week.^2^, ^3^, ^4^ The prevalence of NE has been associated with the male gender, long‐term disposable diaper use, mental stress, and poverty.^2^, ^5^, ^6^

Nocturnal polyuria in conjunction with an abnormal circadian release of antidiuretic hormone or arginine vasopressin is a major contributing factor to NE.^7^ The other etiological factors are high arousal threshold, low functional bladder capacity, detrusor overactivity (DO), and reduced bladder sensation, and lack of inhibition of the micturition reflex. These developmental disorders are influenced by both genetic and environmental factors. The cause of lower urinary tract symptoms and disorders can vary according to the symptoms presented (urge, voiding postponement, dysfunctional voiding).^8^, ^9^ The diagnostic process consists of medical history, a clinical examination, frequency‐volume charts and appropriate investigations.^10^ In most cases, urodynamic studies (UDS) are unnecessary.^11^ UDS is generally performed in children with neurogenic bladders, sphincter dysfunctions, anorectal malformations, voiding dysfunction including urge syndrome and underactive bladder, vesicoureteral reflux, urinary incontinence, infravesical obstruction, and obstructive uropathy.^12^ Since UDS can detect abnormalities in a small proportion of enuretics, it should be carefully selected.^11^

Diagnostic imaging and UDS are recommended for children with significant daytime symptoms, urinary tract infections (UTI), structural renal dysfunction, or cases that are resistant to treatment.^13^ The results of UDSs on patients with primary enuresis are typically indicative of overactive detrusors or diminished bladder functions and compliance.^14^

While several researchers have demonstrated the value of urodynamic evaluations in children with severe non‐monosymptomatic nocturnal enuresis (NMNE) or therapy‐resistant NE, the effectiveness of UDS evaluations in managing NE and true bladder function remains unclear.^15^, ^16^ Accordingly, this study aimed to assess the urodynamics of consecutive patients with primary enuresis who were referred to our tertiary referral center.

## 2 MATERIAL AND METHODS

This cross‐sectional study on patients who suffered from enuresis was conducted at Labbafinejad hospital during March 2013 to August 2022.

A questionnaire was used to obtain baseline parameters for toilet training, along with a detailed medical history including bladder and bowel habits and previous medical treatment. Upon taking a detailed individual history, every participant had a physical examination, urine analysis, uroflowmetry with residual urine measurement, and UDS performed and interpreted by an experienced urologist (F.S.). Constipation was defined by ROME IV criteria.^17^

Inclusion criteria were children aged 5−18 years and diagnosed with refractory enuresis according to ICCS guidelines (i.e., no improvement of enuretic episodes or a reduction of less than 50% from baseline after at least 6 months of continuous treatment with good compliance). As part of the treatment, imipramine, anticholinergic drugs, desmopressin, enuretic alarms, and bladder training were used. As part of the study, drugs used in the study were discontinued 72 h before UDS to reduce their effect on urodynamic parameters. Study participants were excluded if they had urologic or history of neurogenic lesions (myelodysplasia, spinal cord disorders, and mental retardation), diabetes mellitus, family history of enuresis, current UTIs, or nocturnal polyuria.

All patients underwent UDS according to a standardized protocol, which was developed in accordance with the urodynamic guidelines of the International Continence Society (ICS). A Medical Measurement Systems urodynamic machine was used for the entire study group. We used a 5‐F Double P‐lumen urodynamic catheter for saline infusion at room temperature at a rate ranging between 10 and 50 mL/min, depending on the patient's age. EMGs were performed using peri‐anal surface electrodes. Cystometric capacity was categorized according to its relation to age as low, high, and normal. Normal bladder capacity was determined by Koff's formula,^18^ (age in years +2) *30, up to the age of 12 years. Cystometric capacity was considered low if it was below 70% of the expected volume, and high if it was above 150%. A root square of the voided volume was used to estimate Maximum flow rate (Qmax). Detrusor pressure of greater than 30 cmH_2_O during the voiding phase was considered high. Bladder compliance was considered normal if it was 10 cmH_2_O at bladder capacity, or 5% of normal capacity per cmH_2_O, or approximately 20 cmH_2_O at expected bladder capacity.^19^, ^20^ Residual volumes (PVR) over 30 mL or 21% of bladder capacity in children between 4 and 6 years old, over 20 mL or 15% of bladder capacity in children between 7 and 12 years old and over 50 mL, following double voiding in children older than 12 years were considered abnormally high.^8^ Involuntary detrusor contractions > 15 cmH_2_O during the filling phase were defined as DO.^20^

All procedures involving human participants in the study were approved by the Ethics Committee of the Urology and Nephrology Research Center of Shahid Beheshti University of Medical Sciences with number IR.SBMU.UNRC.REC.1401.025. Written informed consent was signed by the parents or legal guardians of all children, outlining the aim, benefits, and potential adverse effects of the study. Also, this is under the Helsinki declaration of 1964 and its later amendments.

The Statistical Package for Social Sciences IBM (SPSS‐IBM), version 26 (SPSS Inc.) was used to perform the analysis after the data had been double‐checked. All statistical analyses were carried out using this software. Quantitative variables were used to describe the Mean and the standard deviation. Qualitative variables were also defined based on numbers and percentages. Intergroup comparisons were made using the *χ* ^2^ test and Fisher's exact test. A *p* < 0.05 was considered statistically significant.

## 3 RESULTS

Of 143 participants, there were 96 children who met the inclusion criteria and enrolled in the study. Participants included 44 boys and 52 girls, of which 41 (38.8%) were diagnosed with MNE and 55 (61.2%) with NMNE. Children's ages ranged from 5 to 18 years (mean: 9.32 ± 3.80 years in those with MNE and 9.90 ± 4.08 years in those with NMNE) (Table 1).

**Table 1:** Demographic and urodynamic parameter of participants.

	MNE	NMNE	Total
*N* (%)	41 (38.8)	55 (61.2)	96 (100)
Age (mean ± SD)	9.3 ± 3.8	9.9 ± 4.0	9.6 ± 3.9
Female:Male	21:20	31:24	52:44
*Urodynamic findings (mean ± SD)*			
Bladder capacity	208.8 ± 102.1	228.1 ± 113.7	220.0 ± 108.8
Bladder compliance	24.7 ± 14.6	19.5 ± 14.1	21.6 ± 14.5
PVR	20.4 ± 26.7	57.5 ± 84.1	41.6 ± 68.3
Q‐max	18.3 ± 8.4	16.2 ± 28.8	17.1 ± 22.3
Abnormal urodynamic	40 (97.5)	50 (90.9)	90 (93.8)
DO	31 (75.6)	39 (70.9)	70 (72.9)
Low bladder capacity	14 (34.1)	21 (38.1)	35 (36.5)
Low compliance	15 (36.5)	28 (50.9)	43 (44.7)
Increased PVR	16 (39.0)	39 (70.9)	76 (79.2)
Abnormal voiding pattern	20 (48.7)	42 (76.3)	62 (64.5)
DSD	3 (7.3)	2 (3.6)	5 (5.2)
Normal urodynamic	1 (2.4)	5 (9.0)	6 (6.3)

Abbreviations: DO, detrusor overactivity; DSD, detrusor sphincter dyssynergia; MNE, monosymptomatic nocturnal enuresis; NMNE, nonmonosymptomatic nocturnal; PVR, postvoiding residual volume; SD, standard deviation.

Urodynamic analyses were found to be normal in 6 patients (6.3%). DO was identified in 70 (72.9%) patients. Bladder capacity was lower than optimum in 35 (36.5%) patients. Bladder compliance was low in 43 patients. Both DO and lower bladder capacity were found in 22 (22.9%) patients. Increased PVR were found in 76 (79.2%). The mean bladder compliance was 21.66 ± 14.52 mL/cmH_2_O (2−75 cmH_2_O) (Table 1).

Of the 55 children with NMNE, 50 (90.9%) had abnormal urodynamic findings. 39 (70.9%) had DO, 28 (50.9%) had low compliance,39(70.9%) had increased PVR and 2 (3.6%) had detrusor sphincter dyssynergia. Of the 41 children with MNE, 40 (97.5%) had abnormal urodynamic findings, and out of these children, 31 (75.6%) had DO, 15 (36.5%) had low compliance, 16 (39.0%) had increased PVR and 3 (7.3%) had detrusor sphincter dyssynergia. Five children with NMNE and one child with MNE had normal urodynamic findings. The difference was not statistically different (*p* = 0.23) (Table 1).

Interpattern *χ* ^2^ analysis was done to determine the relationship between urinary symptoms (obstructive and irritative urinary symptoms) and the urodynamic findings. Obstructive urinary symptoms were significantly related to both increased PVR and DO. NMNE was significantly related to increased PVR (Table 2).

**Table 2:** Inter‐pattern comparison of abnormal urodynamic findings with obstructive and irritative urinary symptoms.

Abnormal urodynamic findings (*n*)	Obstructive symptoms		*p* Value	Irritative symptoms		*p* Value	NMNE		*p* Value
	Yes *n* (%)	No *n* (%)		Yes *n* (%)	No *n* (%)		Yes *n* (%)	No *n* (%)	
Low capacity	9 (18)	26 (14.9)	0.573	16 (15.8)	19 (15.4)	0.076	21 (38.1)	14 (34.1)	0.684
Low compliance	13 (26)	30 (17.2)	0.836	28 (27.7)	15 (12.1)	0.163	28 (50.9)	15 (36.5)	0.163
DO	16 (32)	54 (31)	**0.026**	28 (27.7)	42 (34.1)	0.057	39 (70.9)	31 (75.6)	0.608
Increased PVR	12 (24)	64 (37.9)	**0.001**	29 (28.7)	47 (38.2)	0.128	39 (70.9)	37 (90.2)	**0.021**

Abbreviations: DO, detrusor overactivity; PVR, postvoiding residual volume.

There was a statistically significant relation between NMNE and abnormal voiding pattern (*p* = 0.005). Specific urinary symptoms, history of UTI and bowel habits were demonstrated in Table 3. No significant association was found between the history of UTI and constipation with UDS abnormality (*p* = 1.0 and *p* = 0.49 respectively) (Table 3).

**Table 3:** Urinary symptoms and bowel habits of participants.

	MNE *n* (%)	NMNE *n* (%)	Total *N* (%)
*Urinary symptoms*			
Frequency	0 (0)	32 (58.2)	32 (33.3)
Urgency	0 (0)	2 (3.6)	2 (2.0)
Incontinency	21 (51.2)	39 (70.9)	60 (62.5)
Strain	0 (0)	16 (29.0)	16 (16.6)
Intermittency	0 (0)	2 (3.6)	2 (2.0)
Dribbling	0 (0)	0 (0)	0 (0)
Incomplete voiding	0 (0)	16 (29.0)	16 (16.6)
Weak stream	0 (0)	4 (7.27)	4 (4.1)
Dysuria	0 (0)	1 (1.8)	1 (1.0)
Nocturnal polyuria	5 (12.1)	15 (27.2)	20 (20.8)
UTI	5 (12.1)	5 (9.0)	10 (1.0)
*Bowel habits*			
Encopresis	0 (0)	3 (5.4)	3 (3.1)
Constipation	4 (9.7)	6 (10.9)	10 (1.0)

Abbreviations: MNE, monosymptomatic nocturnal enuresis; NMNE, nonmonosymptomatic nocturnal enuresis; UTI, urinary tract infection.

## 4 DISCUSSION

In the current study, several urodynamic findings were presented and combinations were investigated to explain bladder functional disorders in enuretic patients and identify parameters that may be indicative of bladder dysfunction. Our results showed that, 97.6% of the patients with MNE and 90.9% of patients with NMNE had abnormal urodynamic findings, and significant correlations were observed between obstructive urinary symptoms with DO and increased PVR. There have been several reports of an association between enuresis and bladder dysfunction. In which small urine volume, reduced capacity, and overactive detrusors have been the most significant urodynamic findings,^11^, ^12^, ^21^ According to our results, the incidence of abnormal urodynamic results was 97.5% in MNE patients while it was 90.9% in NMNE patients.

In a study conducted by Naseri et al., 63% of participants had overactive detrusors, and 80% had a small capacity bladder. The study also found abnormal UDS in 85% of NMNE patients and 41.2% of MNE patients, which was not statistically significant.^22^ Another study conducted by the author found that abnormal urodynamic findings were present in 53% of patients with MNE, as well as 43% of patients with NMNE, which suggests that abnormal UDS is as common in both conditions.^16^

In Tolunay et al.'s study only four out of 27 patients with NMNE had normal urodynamic results (7.4%), while 16 (59.3%) had overactive detrusors.^18^ According to Jung et al., more than half of their NMNE patients who had preoperative diagnoses of overactive bladder (OAB) or dysfunctional voiding had different urodynamic diagnosis.^23^ Considering that the treatment of NMNE involves treating both lower urinary tract symptoms (LUTS) and enuresis, it is important to note that LUTS involves heterogeneous urodynamic abnormalities. Thus, LUTS should be defined and treated as per individual patterns. Furthermore, several studies have shown that a significant proportion of children with refractory MNE have bladder reservoir dysfunction during night sleep despite having normal daytime urodynamics and maximal voided volume.^19^, ^20^ Nevertheless, many of the current management approaches are nonspecific and noninvasive. According to the European Bladder Dysfunction Study, urodynamic results were inconsistent with clinical diagnoses of either OAB or DV, however, treatment aimed at either condition showed cross‐efficacy. The current management systems, which we acknowledge as highly useful in most cases, are supported by this redundancy in diagnosis and treatment. Nevertheless, refractory cases like those described here may require accurate diagnosis and tailored treatment.^24^ The discrepancy between clinical features and UDS could explain some instances of treatment failure. Contrary to previous negative statements regarding the utility of UDS in the treatment of patients with refractory enuresis, most of the patients included in this study manifested at least one abnormal finding, demonstrating that UDS may be helpful in such patients.

In our study DO and increased PVR were the most common urodynamic findings, with the latter showing statistical significance with NMNE. There was a higher incidence of NMNE in girls in our study, and urinary incontinence was the most prevalent daily urinary symptom (Table 3). Studies have found that bedwetting is more common among boys, and urinary incontinence more common among girls.^25^

A high incidence of constipation has been reported among patients with enuresis, which was traditionally thought to be a risk factor for enuresis.^26^ While functional constipation treatment has been shown to positively affect LUTS/enuresis, many studies have failed to demonstrate this association and have revealed similar prevalence irrespective of enuresis.^27^ Approximately 20% of children in our study experienced constipation, which is similar to the percentage reported in Fujitani et al.'s recent study on constipation epidemiology.^28^ Our analysis did not detect a significant association; especially when considering children with ME alone, as we found that ME was less prevalent in children with constipation.

According to a recent study, unlike MNE, lower urinary tract dysfunction and NMNE were correlated with constipation.^23^ The association between MNE and constipation previously postulated in the literature may have resulted from the lack of distinction between MNE and NMNE, since the latter is frequently associated with constipation. Consequently, constipation might not be a risk factor for MNE, but may be associated with lower urinary tract dysfunction and therefore may contribute to NMNE (Table 3).

Prior research has demonstrated a link between a history of UTI and NE.^29^ However, the exact mechanisms for the association between a history of UTI and OAB remain unclear. A UTI, particularly cystitis, is known to cause involuntary DO.^30^ While UTIs can be treated, the symptoms of DO may persist and negatively affect the bladder's function.^31^ In our study 10 out of 90 children with UDS abnormality had history of recurrent UTI, but there was no significant correlation in between.

This study was limited in the number of patients and retrospective design, so future studies should confirm our conclusions. A distinction should be made between children with primary and secondary NMNE. The UDS protocol and methodology were strictly controlled throughout the study, and the interpretation of the urodynamic findings remained constant.

## 5 CONCLUSION

The present study revealed a notable prevalence of bladder function disorders among refractory MNE and NMNE patients, characterized by diminished functional capacity, reduced bladder compliance, and pronounced DO. Notably, nocturnal enuresis may manifest as the sole symptom, yet its association with bladder overactivity, UTIs, and constipation due to pelvic floor overactivity should be noted. These urodynamic findings offer valuable insights for guiding the assessment and tailored treatment of children grappling with primary refractory nocturnal enuresis.

## AUTHOR CONTRIBUTIONS

**Farzaneh Sharifiaghdas**: Conceptualization; methodology; project administration; supervision; writing—original draft; writing—review and editing. **Behzad Narouie**: Data curation; formal analysis; investigation; supervision; writing—review and editing. **Mohadese Ahmadzade**: Data curation; formal analysis; investigation; methodology; writing—original draft; writing—review and editing. **Hamidreza Rouientan**: Data curation; formal analysis; investigation; methodology; validation; writing—original draft; writing—review and editing. **Darya Najafi**: Data curation; formal analysis; investigation; writing—original draft. **Mehdi Dadpour**: Data curation; supervision; writing—review and editing. **Nariman Latifi**: Data curation; investigation; writing—original draft. **Hamideh Hanafi Bojd**: Data curation; formal analysis; validation; writing—review and editing. **Sobhan Sabzi**: Data curation; investigation; methodology; supervision; writing—review and editing.

## CONFLICT OF INTEREST STATEMENT

The authors declare no conflict of interest.

## TRANSPARENCY STATEMENT

The lead author Farzaneh Sharifiaghdas affirms that this manuscript is an honest, accurate, and transparent account of the study being reported; that no important aspects of the study have been omitted; and that any discrepancies from the study as planned (and, if relevant, registered) have been explained.

## Data Availability

The data that support the findings of this study are available from the corresponding author upon reasonable request.
